# Comparison of the effectiveness of manual massage, long-wave diathermy, and sham long-wave diathermy for the management of delayed-onset muscle soreness: a randomized controlled trial

**DOI:** 10.1186/s40945-019-0073-4

**Published:** 2020-01-15

**Authors:** Lorenzo Visconti, Corrado Forni, Rudi Coser, Marco Trucco, Elisa Magnano, Gianpiero Capra

**Affiliations:** 1 Studi fisioterapici di Montagna, Rue Palleusieux 1 11010 Pré-Saint-Didier, Aosta, Italy; 2 Azienda Ospedaliera Maggiore della carità, Novara, Italy; 3 Studi fisioterapici di Montagna, Trento, Italy; 4 Istituto clinico San Camillo, Torino, Italy; 5 Università Torino Bachelor in Physiotherapy, Torino, Italy; 6 Supsi Scuola Universitaria per la Svizzera Italiana, Lugano, Switzerland

**Keywords:** DOMS, Lower extremity, Long-wave diathermy, Manual massage

## Abstract

**Background:**

Delayed-onset muscle soreness (DOMS) is a specific symptom that typically arises after unaccustomed eccentric muscular effort. It increases typically 24–72 h post-exercise and can affect physical performance. The pathophysiology of DOMS remains unclear, although it seems to be related to the remodeling phase of myofibrils. Different types of treatments have been proposed to minimize DOMS after exercise; however, no clear gold standard treatment exists. Among the most popular and easy-to-apply treatments, manual massage is often performed by clinicians and has been documented to be effective in reducing symptoms. For several years, long-wave diathermy (LWD) has been performed to manage musculoskeletal complaints, such as DOMS; however, no studies have reported its efficacy thus far.

This study aimed to compare the clinical effectiveness of LWD, sham LWD, and manual massage in participants with lower limb DOMS.

**Methods:**

Participants with lower limb DOMS were included in the study. They were randomly assigned to undergo real LWD, sham LWD, or manual massage. The Numeric Pain Rating Scale (NPRS) score was the primary outcome, and the Patient Global Impression of Change (PGIC) Scale score was the secondary outcome. Outcomes were collected before and immediately after the treatment. Analysis of variance was performed to compare the post-treatment NPRS value variability among the groups and to compare the pre- and post-treatment NPRS differences among the groups.

**Results:**

No clinically relevant differences were observed regarding the NPRS value variability among real LWD, sham LWD and manual massage groups. Differences were observed in the PGIC Scale scores.

**Conclusions:**

Future studies are needed to have a better understanding about the treatment of DOMS in clinical practice.

**Trial registration:**

The trial was registered on 29th February 2016 in ClinicalTrials.gov (NCT02693678).

## Background

Delayed-onset muscle soreness (DOMS) is one of the most common complaints of clinicians working in the sports field [1]. Soreness, which typically occurs 24–72 h post-exercise, is observed in the muscle especially after heavy eccentric exercise [2]. Although DOMS was believed to be caused by sarcolemma injury, the pathophysiology of DOMS remains unclear, and a recent study has highlighted the relationship between DOMS and myofibril remodeling [3]. DOMS has an impact on physical performance, as it affects coordination, muscle strength, and abilities to absorb shock [4, 5]. Different treatments have been proposed to manage DOMS [6]. As the pathophysiology of DOMS is unclear and no clear gold standard treatment has been established for managing DOMS, treatments range from applying heat, cold, compression, and massage [6–8]. As DOMS affects physical performance, it is of utmost importance, especially in the field of professional sports, that athletes can be immediately relieved from DOMS so that they can be trained or can compete with the absence of such conditions. Moreover, it is important for DOMS to be treated using easy-to-perform strategies, as sport teams are often engaged in traveling during competitions.

Manual massage is considered one of the most common and easy-to-perform treatments to relieve DOMS in clinical practice, and many authors have described its applications [9–12]. Moreover, long-wave diathermy (LWD; also known as *capacitive and resistive electric transfer therapy*) has recently received great clinical interest in the field of sports. LWD produces heat and is supposed to improve microcirculation flow and metabolic processes; however, currently, evidence of its presumed induced effect is insufficient. Clinical use of LWD in treating DOMS has been common since 2000. Heat has been suggested to relieve DOMS [8]. Despite the wide use of LWD in clinical practice, since more than a decade, and a recent study on the effect of such treatment on recovery in recreational runners [13], a study that confirms the efficacy of such treatment in DOMS does not exist.

This study aimed to investigate the effects of manual massage, real LWD (rLWD), and sham LWD (sLWD) on pain and its post-treatment effects in a group of athletes presenting with lower limb DOMS.

## Methods

**Fig. 1:**
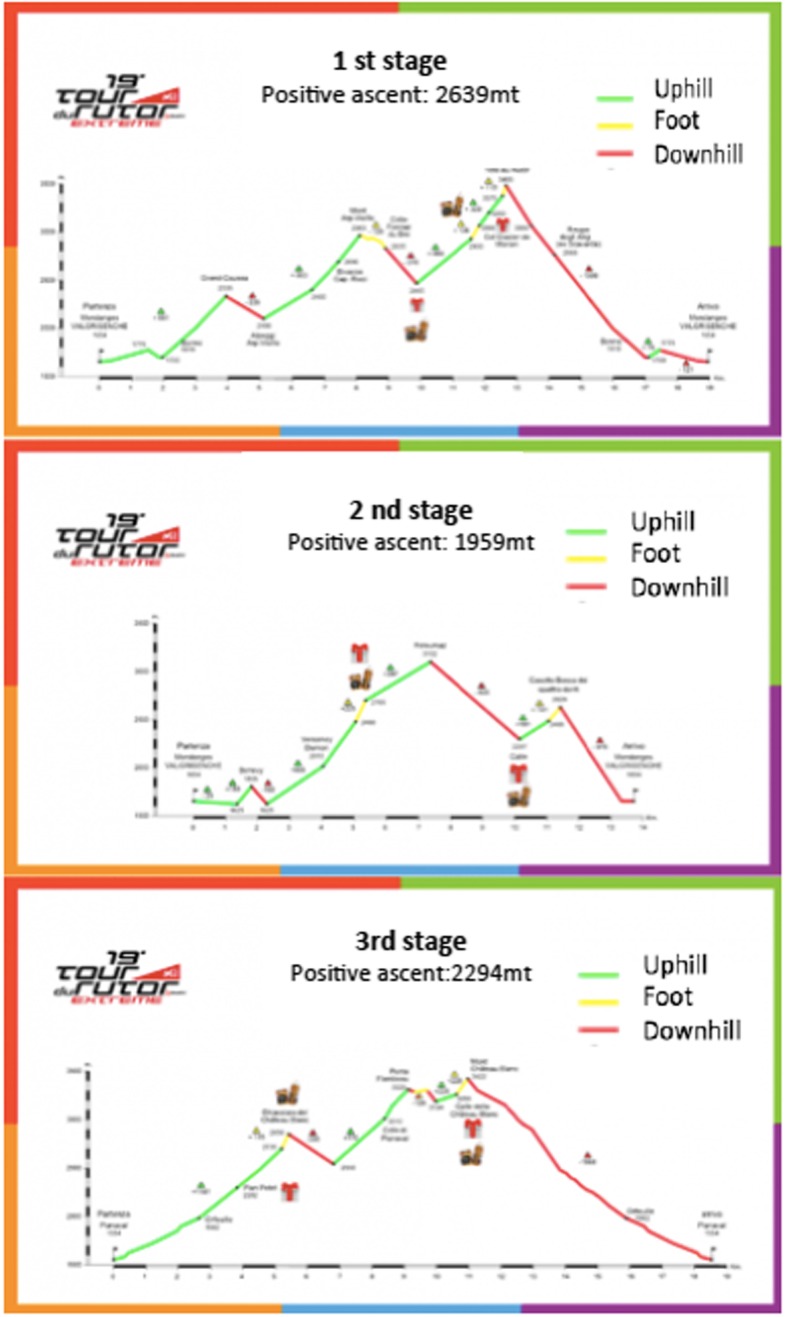
18th Millet Tour du Rutor Extreme stages

In this study, male athletes with lower limb DOMS were recruited. The participants were ski mountaineering racers who participated in a 3-day race, the *18th Millet Tour du Rutor Extreme* (Arvier, Italy). The ski mountaineering alternate uphill phase with downhill free-ride ski exposing the athletes to highly sustained eccentric effort over the 3-days race (Fig. 1). The participants were treated during their rest time between stages while they were experiencing the peak phase of DOMS. The participants were excluded in this experiment if, during assessment, they complained of musculoskeletal or general health problems other than DOMS. Two senior-level physiotherapists, unaware of the group of treatments that participants would be assigned to, assessed the eligibility criteria in the study. The participants were randomly assigned, using an online software program (*random.org*), to the three treatments, with the assignment of treatment conducted by an undergraduate physiotherapy students at the end of their bachelor’s degree. They treated the athletes with lower limb DOMS during its peak phase, from 24 to 72 h post-exercise according to data from the literature [2–10].

Sample size calculation was based on a mean change of 2 points in the Numerical Pain Rating Scale (NPRS) score, which is clinically relevant for musculoskeletal pain [14]. To detect a difference of 2 NPRS points, considering a standard deviation of 1.5, a power 1 − β of 0.8, and a probability of type-I α error of 0.05, 51 participants (17 per group) were required in this experiment.

Consent to perform the study was obtained from the local ethics committee *Azienda Ospedaliera Della Valle d’Aosta* prot. nr 6719 22/1/2015, and all the procedures conformed to the Declaration of Helsinki. All the participants provided informed consent for inclusion in the study. The trial was preventively registered in ClinicalTrials.gov (NCT02693678).

### Treatments

The participants were randomly divided into three groups. The participants allocated to the manual massage group were treated with manual massage as described in another study^7^. A 10-min pain-free *effleurage* was performed in both limbs, with particular emphasis on the areas reported to be symptomatic with DOMS according to the athletes, was performed. The participants lay in the prone position for the treatment of the muscles of the posterior compartment of the lower limb (hamstrings and triceps surae) and in the supine position for the treatment of the muscles of the anterior compartment (quadriceps and foot dorsiflexors).

Participants allocated to the rLWD group were treated with switched-on LWD (Red Coral, Tecnosix, Sixtus, Italy). A 10-min treatment on the symptomatic areas in capacitive mode (750 kHz) was performed. The device producers directly gave the instructions in the performance of the technique.

Participants allocated to the sLWD group were treated with switched-off LWD. The treatment was a 10-min switched off LWD (Red Coral Tecnosix, Sixtus, Italy) on the symptomatic areas. To perform sLWD, the device was switched-on for 10 s to provide warmth and then switched-off for the rest of the treatment session. Participants were unaware that the device was switched-off, and two different operators performed the switching on and off of the LWD device; thus, also the operator performing the treatment was blinded.

To reduce bias caused by negative expectations that have been described to influence pain reports [15], in this study, both rLWD and sLWD groups started the treatment with the operator stating that the device was active.

rLWD, sLWD and manual massage treatments were applied with a neutral cream.

### Outcomes

Each participant in the experiment was asked to answer a NPRS questionnaire before and after receiving the treatments as a primary outcome. The athletes were instructed that NPRS can range from 0 (no pain) to 10 (worst imaginable pain) and then asked to score their pain. The same score was requested immediately after they received the treatment. The NPRS has been provided to be a reliable tool for assessing the pain level in the musculoskeletal population [16].

The participants were also asked to answer a *Patient Global Impression of Change Scale* (PGIC) [17] questionnaire after undergoing treatment. In this seven-item questionnaire, the participants could report to feel the following: “a great deal better,” “much better,” “moderately better,” “somewhat better,” “a little better,” “almost the same,” or to feel “no change or worst” after treatment.

### Data analyses

In this study, data were analyzed using *JASP* 0.8.6 for Mac 2018. An analysis of variance (ANOVA) was performed to compare the variability in post-treatment NPRS values among the groups and in pre- and post-treatment NPRS score differences among the groups, and a *p* value lower than 0.05 was considered statistically significant.

Data were presented as pre- and post-treatment mean NPRS scores and were listed in relation to the frequency on the PGIC Scale. The proportion of reports in the PGIC Scale between the groups was calculated with the Kruskal-Wallis test.

The pre-treatment characteristics and proportions of the participants in the different treatment groups that attained a result equal to or better than the clinically relevant change in NPRS score were analyzed (Table 1).

**Table 1:** Baseline and demographic characteristics of the participants and descriptive statistics. Values are expressed as mean (standard deviation). Δ = difference

	Group		
	sLWD *n* = 19	rLWD *n* = 17	Massage *n* = 19
Age, years	36.6 (6.8)	39.4 (7.4)	40.8 (9.2)
Pre-treatment NPRS score	5.8 (2.2)	5.6 (1.4)	5.7 (2.3)
Post-treatment NPRS score ANOVA, *F* = 023 and *p* = 0.79	3.1 (1.9)	3.3 (1.6)	3.3 (2.1)
Pre-post treatment Δ ANOVA, *F* = 0.08 and *p* = 0.91	2.7 (2.0)	2.3 (1.5)	2.4 (1.2)
Relative proportion attaining a result equal or higher than 2 points in the pre-post treatment NPRS	63,1%	64.7%	68.4%

The association between the PGIC Sale and NPRS scores was calculated using the Kendall tau (*τ*) rank correlation that ranged between − 1 (perfect inversion association) and 0 (absence of association). Values that ranged from 0 to − 0.3 indicated a weak correlation, from − 0.3 to − 0.7 a moderate correlation; and > − 0.7 a strong correlation.

## Results

### Flow of participants

The manual massage group was composed of 19 participants; the rLWD group, 17 participants; and the sLWD group of 19 participants.

The groups were comparable in relation to the demographic and pre-treatment outcome characteristics. The baseline characteristics are shown in Table 1. The participants’ ages ranged from 23 to 60 years (mean, 38.9 ± 7.9 years), and the mean pretreatment NPRS score in the manual massage, rLWD, and sLWD groups were 5.6/10, 5.7/10, and 5.8/10, respectively.

**Fig. 2:**
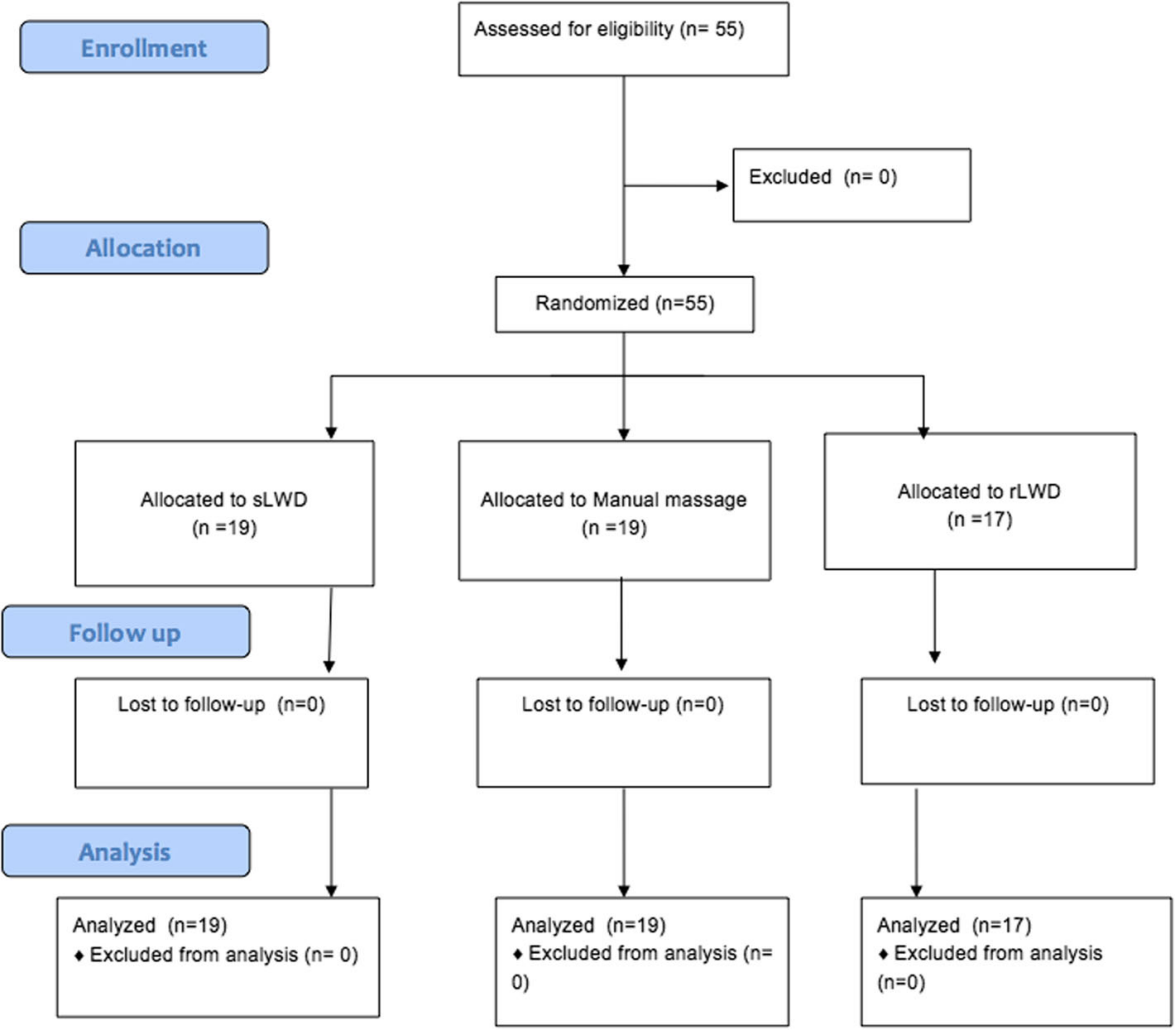
Consort flow diagram

All 55 athletes with lower limb DOMS completed the study (Fig. 2).

### Effects of interventions

**Fig. 3:**
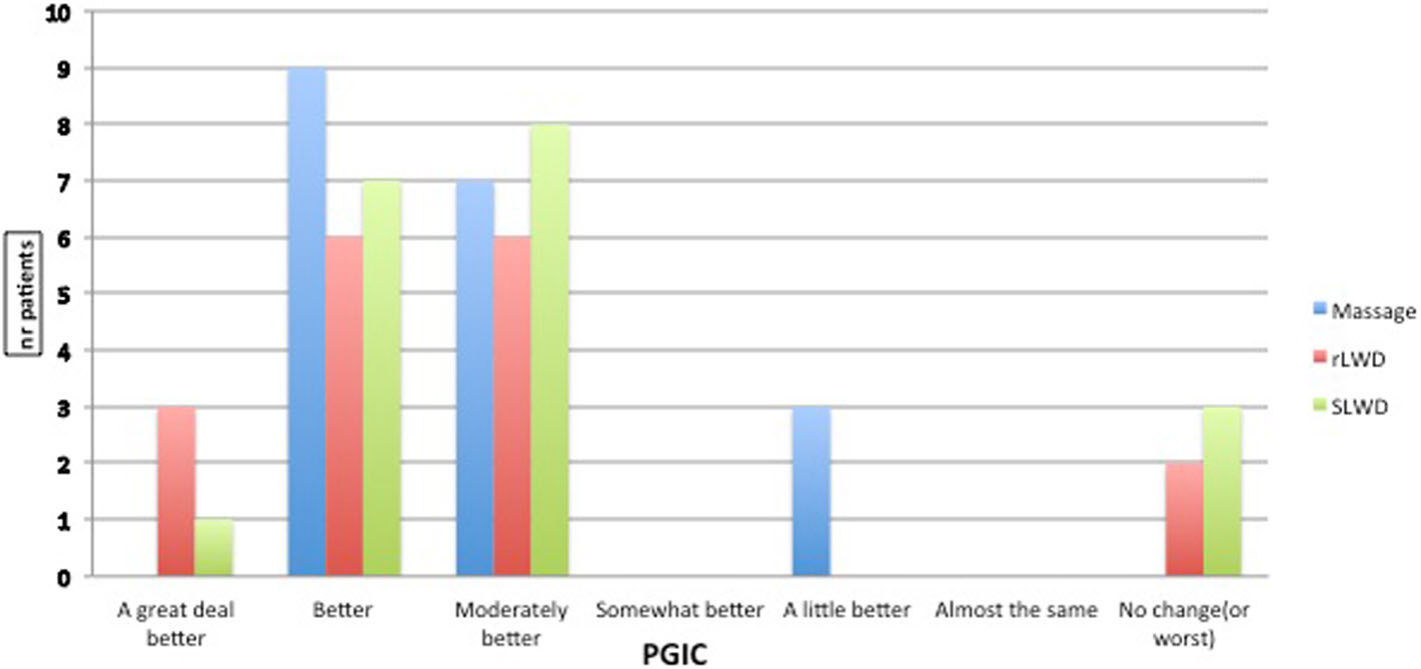
Post-treatment Patient Global Impression of Change Scale scores

The descriptive statistics is shown in Table 1. The ANOVA of the post-treatment NPRS score showed no significant difference between the treatment group variability means (*p* = 0.91 and *F* = 0.08). The ANOVA of the mean between the group variability pre- and post-treatment NPRS scores showed no significant difference (*p* = 0.79 and *F* = 0.23). Differences can be observed in the PGIC frequency reports (Fig. 3), although no significant differences can be found among the groups (Kruskal-Wallis, *p* = 0.638).

The NPRS and PGIC Scale scores demonstrated a moderate correlation, as the Kendall tau rank correlation (*τ*) value was 0.34.

## Discussion

To the best of our knowledge, this is the first randomized controlled trial that compares the effectiveness of real LWD, sham LWD, and manual massage for lower limb DOMS. As outlined, in general, few data on the effects of LWD on musculoskeletal disorders exist in the literature despite a common massive performance of such technique in the clinical setting. As manual massage has been effective for treating DOMS [10, 18–20], we were interested to compare it with LWD and sham LWD. Similar results in DOMS attenuation have been described in high-quality studies (Physiotherapy Evidence Database PEDro score ≥ 6/10) [21] that provided manual massage as a comparable modality to the one described in the present study. In the literature, the effects of electrotherapies on DOMS have been investigated without significant results; however, no studies have considered LWD [22–24]. In this study, although the proportion of participants who attained an improvement of equal or more than NPRS points was higher in the manual massage group (Table 1), similar results were obtained for manual massage, rLWD, and sLWD, considering the post-treatment NPRS score or differences between the pre- and post-treatment NPRS scores. This suggests that the pain reduction reported by athletes with lower limb DOMS in the NPRS may not be attributable to the specific mechanism belonging exclusively to manual massage or LWD.

In the groups treated with LWD (both real or sham), a wider range of post-treatment-reported perceptions measured using the PGIC Scale was referred than in the manual massage group. The authors suggest the possibility that this outcome can be attributable to patient expectations in relation to treatment. Some athletes, both in the real and sham LWD groups, reported feeling “a great deal better” or “no change” or “worst” after treatment in the PGIC Scale, whereas in the manual massage group, the participants generally reported feeling “better” or “moderately better” after receiving the treatment. The literature described how expectations and persuasion can interfere with perceptions of an event, influencing individual output and behavioral responses [25, 26].^.^ It could be of interest to the clinical practitioners to identify the participants who can benefit from the idea of receiving a strongly beneficial perceived treatment.

In the present study, after treatment, interventions as well as sham treatments produced similar results. An improvement of at least 2 points in NPRS score (Table 1) was achieved by 68% of participants receiving manual massage, by 64.7% receiving rLWD and by 63.1% of partecipants receiving sham LWD. This could be interesting for a sports team that is often engaged in traveling, as manual massage which is an easy-to-perform treatment not requiring adjunctive devices other than therapist’s hand results in meaningful improvement in lower limb DOMS related pain. Other treatments such as whole-body cryotherapy and cold water immersion with similar results to the present study on DOMS reduction have been described [27], but these modalities are more demanding from a logistical point of view than manual massage or LWD, especially for those who are engaged in traveling.

## Limitations

This study had several limitations, mainly related to the clinical context in which this data were acquired. Firstly, we did not defined a NPRS score as an inclusion criterion. This could have introduced heterogeneity among included participants; however, this was necessary in order to have the possibility to reach the a-priori calculated sample size. Secondly, we assessed the outcomes immediately after the provision of treatments, at rest, and no follow-up (short or medium term) was performed. This could lead to an overestimation of the treatment effects, as DOMS is typically present during the movement. We made this choice in order not to disturb excessively the athletes involved in the competition. For the same reason, the number of assessed outcome was restricted. In future studies, it would be of interest to include physical outcomes related to DOMS, such as pain on stretch, muscle strength, or other functional outcomes as suggested in other papers [27].

## Conclusions

This study showed that manual massage, rLWD, and sLWD did not led to statistically significant changes in NPRS score of lower limb DOMS in ski mountaineering athletes. Future studies, including further outcomes measures, are justified in order to find more effectiveness treatments for the DOMS.

## Data Availability

Please contact author for data requests.

## Abbreviations

ANOVA: Analysis of variance
DOMS: Delayed-onset muscle soreness
LWD: Long-wave diathermy
NPRS: Numeric pain rating scale
PGIC: Patient global impression of change
SD: Standard deviation
